# Does interviewer gender influence a mother’s response to household surveys about maternal and child health in traditional settings? A qualitative study in Bihar, India

**DOI:** 10.1371/journal.pone.0252120

**Published:** 2021-06-16

**Authors:** Nancy Vollmer, Mansha Singh, Navika Harshe, Joseph J. Valadez

**Affiliations:** Department of International Public Health, Liverpool School of Tropical Medicine, Liverpool, United Kingdom; ESIC Medical College & PGIMSR, INDIA

## Abstract

**Background:**

Two probability surveys, conducted in the same districts of Bihar, India (Aurangabad and Gopalganj) at approximately the same time in 2016 using identical questionnaires and similar survey methods, produced significantly different responses for 37.2% (58/156) of the indicator comparisons. Interviewers for one survey were men while for the other they were women. Respondents were mothers of children aged 0–59 months living in a traditional rural setting. We examined the influence of interviewer gender on mothers’ survey responses and their implications for interpreting survey results.

**Methods:**

We used qualitative methods including 10 focus group discussions (FGDs) and 33 in-depth interviews (IDIs) in the same locations as the 2016 surveys. FGD participants were purposefully selected mothers with children 0–59 months, husbands and other in-law family members. IDIs were carried out with frontline health-workers, enumerators and supervisors from the two previous household surveys.

**Results:**

Findings revealed a preference for female interviewers for household surveys in study districts as they facilitated access to mothers and reduced their discomfort as survey participants. However, this gender preference was related to the survey question. Regardless of age, caste and educational level, most mothers were not permitted to communicate with men (aside from husbands) about female-specific health topics, including birth preparedness, delivery, menstrual cycles, contraception, breastfeeding, sexual behaviour, sexually transmitted disease, and domestic violence. Mothers in higher castes perceived these social restrictions more acutely than mothers in lower castes. There was no systematic direction of the resulting error. Mothers were willing to discuss child health issues with interviewers of either gender.

**Conclusions:**

Interviewer gender is an important consideration when designing survey protocols for maternal and reproductive health studies and when selecting and training enumerators. Female interviewers are optimal for traditional settings in Bihar as they are more likely to obtain accurate data on sensitive topics and reduce the potential for non-sampling error due to their reduced social distance with maternal respondents.

## Introduction

An increasing awareness of the effects that cultural settings have on public health research and practice has led to a growing interest in understanding whether sociodemographic characteristics of interviewers affect the accuracy of survey data [1]. Considerable debate focuses on whether matching interviewer and respondent characteristics improves the accuracy of interview data [2, 3]. This concern is particularly relevant in household surveys that obtain self-reported information about sensitive topics through face-to-face interviews or in surveys that target respondents from marginalized populations [1, 4, 5].

The principal concern is whether observable characteristics of an interviewer (e.g., race, gender, age) or their perceived social status can bias responses to survey questions [1]. The literature considers three types of interviewer effects in survey research: non-participation (refusal to participate), nonresponse (refusal to answer a question) and response quality (accuracy of the response) [3, 4]. Methodological research carried out in high-income countries has focused on item nonresponse and strategies to control response bias using improved survey design and data analysis techniques [6]. Most studies of nonresponse consider interviewer or respondent characteristics alone. Few studies investigate the rapport between interviewer and respondent [7], and fewer still concern research in low resource settings [1, 6].

While it may seem logical that matching interviewer and respondent should reduce social distance and the potential for response error, evidence of interviewer effects across studies on response rates and response quality are inconsistent [1, 2, 5–9]. Several methodological studies show that interviewer effects vary by country and geographic area; however, little is known how cultural norms or social context influence the interviewer-respondent rapport and lead to measurable interviewer effects [1, 3, 4, 10, 11]. Few qualitative studies have explored respondents’ perceptions of the interview process, in particular the acceptability of an interviewer’s gender or how social norms influence female respondent behaviour and survey responses [12]. Such research is particularly lacking for traditional, hierarchical societies such as India, where gender and social norms often govern women’s decision making [13–15]. While some studies in India have investigated interviewer effects, few have considered the influence of gender in household surveys [16]. A study in Nepal revealed a pattern of under-reporting of selected family planning behaviours gathered by male interviewers in traditional communities [13]. However, there is limited available evidence from India on the effect of an interviewer’s gender on responses to household surveys assessing knowledge, attitudes and practices of mothers regarding maternal, newborn and child health (MNCH) services. Our research helps to fill this gap.

In September 2016, two identical MNCH household surveys were carried out by two different organisations in two rural districts of Bihar, India: Gopalganj which is located in the north of Bihar and Aurangabad in the south. The first survey was part of an NGO’s regular programme monitoring; the second one was a separate Third Party Monitoring of the NGO’s activities and the quality of its survey which was requested by the donor [17]. These were the sole objectives of the second survey. In addition to being located in the same districts at similar times, the surveys used identical questionnaires and similar sampling techniques. Respondents for both surveys were mothers of children aged 0–59 months. Rather than producing similar results, the two surveys displayed significant differences in over one-third of 156 comparisons of the indicators which assessed antenatal care (ANC) and birth preparedness, childhood nutrition, child immunization, frontline worker (FLW) support to mothers for breastfeeding, and family planning use [18].

The statistical differences between the two surveys are documented elsewhere [18] and reported among our results. While otherwise identical, the two surveys had one important difference that was not heretofore investigated, namely, the gender of interviewers differed between the two data collection teams. One survey team used all-male data collectors supported by male supervisors while the other had a team of all female data collectors supported by a mix of male and female supervisors. This situation provided an ideal opportunity to explore sociocultural norms regarding participation in household surveys in a rural setting in India and the influence of gender roles on survey responses, including question avoidance and accuracy.

We conducted a qualitative study from December 2017–July 2018 in Aurangabad and Gopalganj districts in Bihar, the same two districts where the twin household surveys had been implemented in 2016. Our objective was to explore the effects of interviewer gender on the interviewing process in the two districts where the quantitative surveys had been carried out. Both are primarily rural districts with a mix of upper caste and lower caste Hindu residents, and minority Muslim communities. Located in northeast India, Bihar is one of the country’s poorest and most densely populated states (N = 110million). It has some of the weakest maternal and child health and nutrition indicators in India [12, 19]. Bihar is a highly patriarchal traditional setting, characterised by low female literacy and autonomy, limited female agency in household decisions, poor spousal communication and low access to social support for married women [20]. Restrictive social norms perpetuate a chronic pattern of unequal gender roles and behaviours. Gender norms support male authoritarian roles, marginalize women’s voice and influence, associate male honour with a woman’s purity and condone male violence toward women [21]. Gender-based violence is pervasive in Bihar which has India’s highest rate of reported domestic violence [22].

Our principal research questions are: How do social norms (cf [23]) affect a mother’s willing participation in household surveys in two rural districts in Bihar, India? How do gender roles influence her ability to respond and the accuracy of her responses to survey questions on MNCH topics? We initially explore the perceived acceptability of male and female interviewers for household surveys on MNCH topics that target mothers of young children. Then we explore how prevailing gender roles and social constraints may influence their survey participation, nonresponse and response quality. Finally, we discuss implications of our findings for future household surveys. We identify survey content that is susceptible to interviewer gender effects and may influence data accuracy and we interpret the discrepancy in the results of the two 2016 surveys.

## Methods

We collected qualitative data using focus group discussions (FGD) and in-depth individual interviews (IDI) with a range of participants to explore perceptions, beliefs and experiences related to gender and social norms in two districts in Bihar. FGDs were conducted with mothers and significant family members, including husbands, mothers-in-law and grandparents. In a few cases (in particular, with upper caste mothers), participants refused to join the FGD but agreed to an IDI instead. Other IDI participants included frontline workers (FLW) who are Accredited Social Health Activists (ASHA) and Anganwadi Workers (AWW). We also conducted in-depth telephone interviews with supervisors and enumerators involved in the earlier household surveys in the same two districts.

Using semi-structured topic guides, we explored how sociocultural norms and processes affect a mother’s participation in household surveys and her ability to respond accurately to survey questions asked by an interviewer of the same or opposite gender. Interview questions focused most intensely on family planning, antenatal care, birth planning, childhood nutrition, and childhood immunizations as these were the indicators with substantial differences between the two household surveys carried out previously in Bihar. We also asked questions to understand reasons why the two surveys agreed on other indicators. The data collection tools, which were developed for this study, can be found in the S1 File. They include Focus Group Discussion Guides for Mothers of Children < 5 Years and Household members (husbands, grandparents, other in-laws living in households with mothers of children < 5 years); and Individual Interview Guides for ASHA/AWW and Interviewers/Supervisors from the prior probability surveys in Bihar (see S1 File).

### Sampling

We purposefully selected four villages (two each in Aurangabad and Gopalganj districts) using the sampling frame from the 2016 household surveys comprising a list of all villages and the approximate population sizes. Subsequently the field team purposefully selected 45 mothers for five FGDs. The inclusion criteria were residence in Bihar for at least 6-months and having a child under five years old. A second purposive sample of 52 household members was selected for a separate set of five FGDs. These individuals were adult family members living in the same household as a mother selected for interview. Male respondents included husbands or other male family members (e.g., brother-in-law, father-in-law, grandfather). Female respondents included family members such as mother-in-law, sister-in-law or grandmother. FDGs for male and female participants were run separately. Eleven individuals (10 mothers, 1 husband) selected for an FDG declined to participate but agreed to an IDI instead. Eleven frontline workers (FLW) working in the two study districts were recruited for IDIs using convenience sampling (based on availability at the time the research team was present in the study district). Lastly, four enumerators and seven supervisors (male and female) from past household surveys in Bihar were purposefully selected for IDIs carried out by phone. All eligible study participants were conversant in Hindi or English and were over 18 years of age (Table 1).

**Table 1:** Sample size by type of participant and interview method.

Respondent Type	FGD		IDI		Total
	Female	Male	Female	Male	
Mothers of children < 5	45	0	10	0	55
Family members	31	21	0	1	53
FLW (ASHA and AWW)	0	0	11	0	11
Enumerators*	0	0	2	2	4
Supervisors*	0	0	3	4	7
Total	76	21	26	7	130

*from household surveys carried out previously in Bihar.

So that our sample reflected the diversity in the population, the field team developed community maps in each sampled village in both districts with the help of the local AWW. These maps revealed the geographical distribution of different caste groups and the location of marginal communities in each village. The field team used the maps and existing household lists to purposively select respondents from different castes, social groups and locations within the villages to ensure the sample accommodated multiple voices from different economic groups. Selection intended to include marginalized people whose opinions often are not sought, such as the poorest, young mothers, scheduled castes, and minority religions. Economic groups tended to live in the same enclaves within villages. Using community maps to guide the purposive sampling ensured that mothers from the poorest and wealthier groups were included. However, we did not ask for within-caste distinctions so as to maintain the integrity of the selected focus groups. Our choices were verified by the AWW.

### Data collection

The study team comprised a bilingual (Hindi-English) Indian field team which included an experienced research manager, a trained qualitative researcher, and a research assistant supported by two experienced senior researchers. We developed semi-structured interview guides drawing on published literature about interview effects in household surveys and gender relations in India. The sample sizes for FGDs and IDIs were estimated [24] during the protocol writing stage to satisfy the requirements for ethical approval and to anticipate our costs for data collection. However, the sample estimates were simply a guide as our intent was to carry out data collection until reaching thematic saturation [25, 26]. Throughout data collection, the field team communicated regularly with all authors to discuss emerging themes and data saturation. Information from these sessions was used to decide which topics should be probed during subsequent interviews. The field team carried out all FGDs and IDIs in Hindi and data collection continued until saturation was reached.

All FGDs and IDIs were audio-recorded. Two bilingual research assistants transcribed the audio recordings verbatim in Hindi and then translated the transcripts into English. One bilingual author reviewed all transcripts for accuracy and back translated a sample of 25% for quality assurance. The study team discussed the most appropriate English translation for words that did not have direct English equivalents.

### Analysis

Using the Framework Method [27, 28], we took a combined approach to the thematic analysis of the translated interview transcripts and field notes. Our aim was to explore specific *a priori* issues identified by the project and allow themes to emerge inductively from the accounts of the research participants. We used the interview topic guides to create the initial analytical framework (code book) and any additional issues that emerged from the transcripts were added to the codebook as the analysis progressed. The initial framework was revised several times by the two authors with additional codes and categories until agreement was reached on the working analytical framework which was then used to code the entire dataset. Any disagreements in coding were discussed and resolved with a third author. Subsequently, we manually extracted the coded data from each transcript and summarised these in a series of Excel spreadsheets organized by case and code. Using the spreadsheets, we explored the accounts of all respondents, applying an iterative cross-case and within-case analysis to identify recurring patterns and themes in the data and to formulate our interpretations [27, 29]. We identified words and phrases from the interview narratives as supporting evidence and to provide meaning and context. This approach revealed the major themes and subthemes which were reviewed and vetted by all authors. See S1 Table for a summary of the final themes.

To strengthen data validity the study team applied two levels of triangulation [30]. Data triangulation, which solicits multiple perspectives on the same topic to verify the consistency of participant responses. We also used investigator triangulation by collecting the data in a team followed by meetings at the end of each day to elaborate the field notes and discuss emerging themes amongst the field team and senior research staff. This approach increased the reliability of field notes and observations and improved the team’s critical reflection. Field notes were shared with all team members via daily emails and feedback was incorporated into subsequent interviews.

While designing this study, the team reflected on the potential for bias introduced by the study authors. Our data collection team was all female in an effort to reduce the influence of interviewer gender effects in this study. For the data analysis, two members of the study team served as coders and analysists to reduce researcher bias in the management and analysis of the data. In addition, an experienced qualitative researcher from India who was not involved in the data collection, analysed a subset of the data and confirmed the face validity of the study findings.

### Ethics approval and consent to participate

Ethical approval was received from the Indian Ethics Council (**IEC No: IIPHB-IEC-2016/010)** and from the Liverpool School of Tropical Medicine, Research Ethics Committee (**Research Protocol 17–082)** prior to commencing the study. All participants gave written consent prior to participating in an interview or focus group. Oral consent was taken for phone interviews carried out with survey enumerators and supervisors. All data has been anonymised; responses cannot be associated with a participant.

## Results

### Differences in results of two household surveys

Interviewer gender effects may help explain some of the differences in results that surfaced when comparing the two household surveys carried out in Bihar in 2016. The surveys were implemented in the same two districts during similar timeframes using identical questionnaires and similar probability sampling designs. We compared district coverage estimates for 54 indicators in both survey datasets. Some indicators were measured in multiple child age groups resulting in 156 comparisons of indicators; 58 comparisons (37.2%) differed significantly at the 0.05 level. We use the data collected by female interviewers as the reference (see Table 2).

**Table 2:** Significantly different results from data collected by male interviewers compared with data collected by female interviewers in Bihar, India.

Survey domains	Indicators	Comparisons to female interviewer data	Male interviewer overestimates	Male interviewer underestimates	Totals	
	#	#	# (%)	# (%)	Total % difference	% of all indicators
Childhood nutrition	18	36	4 (11.1)	16 (44.4)	55.5	23%
ANC & birth preparedness	19	38	9 (23.7)	9 (23.7)	47.4	24%
Childhood immunisation	8	16	2 (12.5)	5 (31.3)	43.8	10%
FLW home visits	9	18	0 (0.0)	7 (38.9)	38.9	12%
Family planning	7	14	0 (0.0)	2 (14.3)	14.3	9%
Delivery & Newborn care	6	34	4 (11.8)	0 (0.0)	11.8	22%
Total	78	156	19 (12.2)	39 (25.0)	37.2	100%

The differences between the two datasets were mixed, and go in both directions, indicating a lack of a systematic bias. In 39 (25%) of the comparisons, coverage data collected by male interviewers was significantly lower than those of female interviewers while in 19 (12%) of them, the male interviewer displayed significantly higher results [18]. However, the discordance between the two datasets was not limited to gender sensitive questions as we expected. We anticipated that a team of male interviewers would exhibit underestimations of gender-sensitive topics and no discordance on child-focused topics, relative to the data collected by females. As expected, we found significant *underestimation* of family planning coverage (14.3%) and reported FLW home visits during pregnancy and perinatal period (38.9%). Surprisingly, we also found significant *underestimation* of the two child-focused topics (childhood nutrition (44.4%), immunization (31.3%) and significant *overestimation* of coverage of delivery/newborn care services (11.8%). Also, the coverage results for ANC/birth preparedness were similarly overestimated (23.7%) and underestimated (23.7%).

### Background of maternal participants in the qualitative study

Mothers who participated in this study were between 18–45 years ($\overset{-}{x}=29.5$ years, SD = 3.6) and represented a mix of lower caste (60.9%), upper caste (24.4%) and Muslim (14.6%). Education varied with the majority (53.7%) of mothers having no education or incomplete primary school; 26.8% completed primary school only, while the remaining 19.5% completed secondary school or above. All mothers (except one) in the study were married and had at least one child under 59 months.

### Themes in the qualitative analysis

Four themes emerged in the qualitative data: social expectations around communication and gender roles; acceptability of survey topics; intersection of gender, caste and religion; and other factors that influence survey participation.

#### Social expectations around communication and gender roles

Social expectations of mothers with regard to communication and gender roles were similar across all respondent groups (mothers, family members, FLW and enumerators) in our study. They frequently mentioned the seclusion of mothers and their avoidance of men outside the family or village. Mothers were also expected to respect the communication hierarchies among their in-law relatives and to keep their distance from the senior (older) men in the family. Most mothers willingly acknowledged the need to practice restraint when speaking to men. They often chose to wear a veil around outside men or male relatives, which served not only as a physical barrier but also a symbol of isolation and restraint. These customs are consistent with the social norms that revolve around the practice of “*purdah*,” which is still widespread in rural areas of Bihar [1]. Purdah is the veiling and seclusion of women which usually begins with marriage and entry into the in-law family. It is a complex set of rules that involve restrictions on female activity and autonomy and expectations of female modesty and censure to maintain the honour and harmony of the family [2].

‘‘*It is our traditional value that women show respect by distancing themselves from other ‘outside’ men and this must be adhered to*.”

*[Family member, FGD]*

“*The village women wouldn’t speak to men; for example*, *they talk to men having covered their faces with a veil*. *The veil or parda is not just physical but also in behaviour… women must practice restraint in their behaviour and communication when they speak to men*.”

*[AWW, IDI]*

“*The custom in Bihar is that women don’t speak… not with her male relatives*. *With us*, *there are some hierarchies*, *okay*, *which we have to adhere to*.”

*[Family member, FGD]*

Consistent with these assumed gender roles, mothers in our study area were usually reluctant to communicate with a male in any circumstance. They expressed a sense of inhibition and discomfort about talking openly to men outside of their family. A mother’s sense of *‘sharmeeli’* (shyness) and ‘*sharam’* (embarrassment) were mentioned repeatedly by mothers and FLW as reasons for not talking to men. One mother explained, “*We observe ‘lehaj’*” which is a social norm of maintaining family honour and dignity, an expectation of decorum that obliges mothers to self-censor their behaviour and interact openly only with other women. Respondents in all groups frequently mentioned “*shame*” and “*hesitation*” when asked if a mother could speak to a man from outside the family or village.

“*I talk to only the ASHA worker and no one else*. *Due to my sense of shame*, *I do not talk to anyone outside the house*. *There are one or two people in the neighbourhood; I do not talk to them*, *so now even they don’t talk… I do not talk to men because I feel shy*. *Also*, *I can absolutely not talk to a man who is an outsider*.”

*[Mother, FGD]*

“*There is no sense of shame if a woman is talking to another woman*. *But women in the village will definitely feel ashamed while talking to men*.”

*[ASHA, IDI]*

Some mothers feared the repercussions of breaking these rules and norms of seclusion which risked the possibility of questioning and complaints by their family and the wider community:

“*Women do not talk to men because they fear that they will later be questioned and harassed by family or community members if found speaking to men*.”

*[AWW, IDI]*

“*There are restrictions […] when the women talk to men*, *people start complaining to their families (shikayat)*: *‘you’re a woman*, *you should be talking to women [about such things]*.*’*”

*[Mother, FGD]*

Due to restrictive social norms and patriarchy issues prevalent in our study locations, access to mothers in a survey setting is dependent on family permissions. In a survey setting, female interviewers were thought to play a key role in facilitating access to mothers of young children and reducing potential discomfort among them and their immediate family members. A woman faced fewer obstacles at the household level than a man because she posed no moral threat to the mother’s honour, or by extension, to the family’s social status.

“*For conducting health surveys women will talk to other women openly*. *If a man comes then I can’t talk to him… When it comes to talking to a woman there will be a free conversation and I can share everything they are like our sisters*.”

*[Mother, IDI]*

“*We should have females [interviewers]*. *For example*, *although both genders were working on the polio drive*, *when women started giving the polio [drops]*, *polio ended here*. *This is also because men cannot enter the house*, *but women can go in and do their work*.”

*[AWW, IDI]*

#### Acceptability of survey topics

Our findings suggest the gender mix of the interviewer-respondent dyad determined which survey topics were acceptable for discussion. For example, a female interviewer and female respondent (mother) were allowed to discuss any subject, including child health (such as immunizations, nutrition and current infections) and maternal health issues which are considered to be gender sensitive and private. Topics belonging to this latter category included: *birth preparedness*, *pregnancy and delivery*, *reproductive health*, *menstrual cycles*, *contraceptive use*, *breastfeeding*, *sexually transmitted disease*, *and domestic violence*. Aside from their husbands, mothers were not permitted to talk to other men—including male interviewers—about female-specific health topics. But mothers expressed no concerns about discussing personal, maternal health issues with a female interviewer.

“*A woman will talk freely with a woman about her internal problems*. *They will hesitate when it comes to talking to a man about these personal issues because she can’t talk to any man other than her husband*.”

*[Mother, FGD]*

“*On ANC*, *birth preparedness*, *family planning*, *breastfeeding*, *menstruation*, *STIs [sexually transmitted diseases]*: *If a man is asking the questions*, *they would have problems*, *it would be easier for women…irrespective of the caste or religion*.”

*[Male enumerator, IDI]*

“*Mothers hesitate to talk about deliveries and hidden illnesses (gupt rog) which are sexual and reproductive health related problems*. *For general health issues they don’t have a problem talking to men*.”

*[AWW, IDI]*

Nevertheless, even female interviewers had difficulty addressing family planning methods, especially with mothers from Muslim families. Family planning was generally a forbidden subject among Muslim families which often caused discomfort.

“*They would have discomfort on family planning questions…Hindus didn’t have problems with the questions as much as Muslims*, *who’d ask about these questions*.”

*[Female enumerator, IDI]*

In the case of a male interviewer—female respondent (mother), acceptable topics were limited to questions about child health which had no restrictions; mothers were permitted to discuss child health issues with an interviewer of either gender. On probing why mothers were willing to communicate with a male outsider regarding their children, mothers and FLW indicated that the primary duty to care for their children outweighed the mother’s need to follow social norms. In the case of their children’s health, issues of interviewer gender mattered less, and communication carried fewer social restrictions.

“*When it comes to vaccinations for children*, *women can talk to both men and women about that*, *but they can’t talk about their own private health related problems with men*.”

*[Family member, FGD]*

“*A man comes to inquire after the child*, *obviously the woman will speak to them as needed because the child cannot speak for him/herself*, *hence*, *there aren’t any restrictions on her at such a time*. *If someone sees her*, *they’ll know and say*: *‘she’s talking about her child’*. *There isn’t any complaint about that kind of thing*.”

*[AWW, IDI]*

All respondent groups concurred that mothers were unable and unwilling to respond to questions about female-specific topics to a male interviewer. Likewise, they felt it was not feasible for a male interviewer to pose such questions to a mother. Until interview questions have been vetted by family members (in particular the husband or mother-in-law) a male interviewer may have difficulty gaining entry to a household or permission to communicate with a mother. Respondents mentioned several strategies to avoid or deflect sensitive questions by a male interviewer. For example, in some cases, the interview was allowed to take place, but the *husband*, *brother-in-law or mother-in-law* responded on behalf of the mother. In other cases, the mother responded erroneously or rapidly in an attempt to end the interview.

“*When men survey*, *other men talk to them*, *else the elder women talk to them only*.”

*[Family member, FGD]*

“*One time the man wanted to listen and step in and speak for his wife… He kept trying to say*, *‘I know about her*, *I can answer the questions*.*’*”

*[Female enumerator, IDI]*

“*Women workers are needed to talk to the women here*. *When men come*, *women feel embarrassed and hesitant*. *Women tend to answer men quickly and try to end the conversations*. *But with women—the way we are talking to you openly now—that won’t happen with male workers*.”

*[AWW, IDI]*

“*Women never come to survey—you’re the first women to have come*. *We have men surveyors more often*. *When men come to talk*, *we often don’t know how to talk to them*, *we feel shy and maintain respect (lihaj)*. *If they’re men we will not be able to talk to them so freely*, *if they ask of something*, *we will reply to them with something else*.”

*[Mother, IDI]*

In contrast, a male interviewer and male family member (responding on behalf of the mother) have permission to discuss any survey topic including female-specific issues concerning the mother. However, the quality of the information will suffer as most male relatives cannot answer personal questions about the mother’s health, knowledge or attitudes.

“*There is no issue if a man is interviewing and the woman’s husband responds on her behalf*. *But how will the men be able to answer*, *understand what she is thinking*? *It’s obvious he is controlling her in such a situation…*”

*[Male enumerator, IDI]*

Given the lack of female autonomy and norms of female seclusion, these findings raise questions about the validity of self-reported responses in survey research in our study districts when carried out by male enumerators, particularly regarding a mother’s personal health issues or her sexual and reproductive choices, knowledge and attitudes.

#### Intersection of gender, caste and religion

While we initially set out to investigate interviewer gender effects alone, the issue of caste and religion also emerged as an important theme in our data. Our findings suggest the intersection of caste, religion and gender influences a mother’s ability to participate in a household survey. While social expectations to avoid interaction with men (*purdah*) is true for married women from all castes, it is especially important for upper caste mothers as they are expected to model traditional values and to exhibit socially acceptable (shy) behaviour. We found that upper caste mothers generally preferred to avoid communication with most men, including those inside the family.

“*It brings shame to mothers to talk to other men in the family*.”

*[Upper caste family member, FGD]*

“*There are a lot of houses where if a man comes to the door*, *Indian cultural values dictate that women [of our caste] talking to strange men even outside the house is not very acceptable*. *Similarly*, *if only a woman is there… it’s okay*, *but if it’s a man… then that can be difficult*.”

*[Upper caste family member, IDI]*

Upper caste mothers tended to face more severe social constraints than mothers from lower castes who had more freedom to communicate with male enumerators. For example, in some traditional homes in our study districts, upper caste mothers rarely stepped outside the perimeter of their houses on their own. They were expected to remain veiled and secluded from family in-laws and outside men, in part to protect their honour and in part to show deference to their in-laws which helps to maintain family harmony. These patterns are consistent with seclusion norms in Bihar which limit women’s mobility and communication, but are generally understood to be less restrictive for poor women who need to work outside the home, often as labourers in the fields or in market jobs [1].

“*In Hindus*, *upper caste households they don’t get permission to go outside unless for an emergency*. *In Muslim houses it’s the same …it happens here*, *but in lower castes there’s no restriction*, *she can go anywhere*.”

*[Female enumerator, IDI]*

“*In Bihar*, *the woman is house bound generally*, *and that affected the interviews…*. *Honour can become a problem for some people*. *Mothers aren’t able to give responses*, *they feel shy*. *Upper caste communities seem to struggle to respond*, *more so than lower caste communities*.”

*[Male enumerator, IDI]*

“*Mothers don’t have the freedom to talk to new people*. *This is what it’s like in our country*. *In villages*, *even when they are educated*, *they behave like this*, *this is the culture*. *It’s in every community*, *but in upper castes this is more so the case*.”

*[Female enumerator, IDI]*

Enumerators also reported that more extensive permissions were typically needed from upper caste and Muslim families before allowing the mother to participate in a survey.

“*In all houses*, *we would have to take consent ahead with the head of the household*, *the elders*. *In some upper caste families*, *Banias*, *Brahmins*, *Srivastavas*, *some Muslims we had to get consent from another member of the family [too]*.”

*[Male enumerator, IDI]*

“*With the general and upper castes*, *Muslims*, *and actually with people who were more educated*, *there were more permissions needed*. *With lower caste families there was no issue*, *they [mothers] spoke easily*.”

*[Male enumerator, IDI]*

Our findings also suggest that a mother’s age among higher caste females played an important role in her ability to communicate with a male interviewer. Younger and newly married mothers tended to experience greater social restrictions than older mothers when communicating with outside men about any health topics. Older mothers who had been married for 6 or more years expressed greater confidence and were more likely to question the social norms governing their interactions with men.

“*There were instances in which newly married and new mothers were not allowed to do the interviews*, *mostly in upper caste families…*.*yes*, *in all [upper] castes*, *Rajputs*, *Brahmins*, *Srivastavas*, *etc*, *even getting a child vaccinated*, *the mothers-in-law get it done*, *the new mothers aren’t allowed to step out*, *mostly with women aged 22–28 years*.”

*[AWW, IDI]*

“*When a male interviewer*, *when we visit a new [upper caste] mother*, *within two years she has given birth*, *and their husband is around*, *they do feel hesitant*. *Both the family and the interviewee feel reluctant*.”

*[Male enumerator, IDI]*

“*We speak to our mothers-in law or husbands*, *because we are older and married for [a] long time*. *If they [younger mothers] don’t speak to the people at home*, *who can they talk to*? *They could speak to the mothers- and sisters-in-law*. *Mostly they speak to their husbands*, *but not other men in family like father-in-law*, *elder brother-in-law because that will be wrong*.”

*[Upper caste mother, IDI]*

Consistent with the above findings, we found it challenging to engage upper caste mothers in the current study. Caste was a noticeable barrier to their participation. Some upper caste mothers were unwilling to participate in an FGD that included other participants of a lower caste. Others refused to participate in the FGD because it was held in the AWW centre located in the home of a lower caste woman. Still others declined because they may never physically leave their home. In these cases, we were permitted to carry out individual interviews inside their homes under the watchful eye of an elder family member. As an example, one mother-respondent from an upper caste family, even after 8 years of marriage, could not leave the house. We were allowed to meet her only after her household members were assured that the interview would be conducted by a woman. The permission to enter the house was first obtained from men in the family, then from the mother-in-law and finally the mother.

#### Other factors that influence survey participation

We asked mothers to rank the factors that could Influence their participation and responsiveness in a household survey. In order of preference, they first noted the perceived educational level of the interviewer, followed by their gender (preferably an educated female professional), and thirdly a similar age-bracket but preference regarding age was not uniform. A mother’s education also played an important role in her autonomy and ability to communicate with outside men. Illiterate mothers felt they were not permitted to address any men except their husband, unless the issue was related to her children’s health.

“*If the male enumerator is an educated like you*, *my husband will allow me to talk but if there is a less educated male enumerator*, *my husband will start asking me questions*. *But*, *if there is a female enumerator*, *he will have no problem in sending me*.”

*[Mother, FGD]*

“*I can talk about anything with a woman*. *But women from our village cannot talk to a man*. *Educated person can do it*, *not the illiterate one*. *An educated person can talk anytime; I am not educated so I cannot talk to any man from outside*.”

*[Mother, IDI]*

A final factor was location and privacy of the survey interview. Mothers in our study felt strongly that the interview should be carried out alone, with only the mother and interviewer present, and no men in the immediate vicinity. This was especially important when a household survey covered sensitive topics. Mothers were aware of the duty of *sharam* and feared family pressures to maintain silence.

“*If someone was there*, *the male relatives were standing behind us*, *we wouldn’t have been able to speak…*”

[Mother, FGD]

Among family members, the importance of interviewer characteristics was reversed. Family members gave first preference to the enumerator being a female, second to the education of the enumerator, and third to the location and privacy of the interview. Family members generally preferred the Anganwadi centre as an appropriate place to interact with mothers for a household survey. A neutral space outside the home, the Anganwadi centre is accessible to all women and not under the influence of other household members.

“*First of all*, *women should be available for women to be comfortable*. *Everyone agrees*. *Education comes second… Then place and the presence or absence of other people*. *The place where there are no men is the best place [for an interview] …Other people should not be there*. *The Anganwadi centre is the best*, *it’s the most convenient*.”

*[Family member, FGD]*

While the Anganwadi centre was the preferred interview location for its privacy, family members acknowledged that some mothers, especially upper caste, cannot step outside their homes to visit the centre nor could they speak to a male interviewer in their home. These restrictions draw into question their representation in household surveys.

“*Not everyone can come out*, *the higher castes*. *Some women don’t step out of the house*. *They’d have to be spoken to at home*. *About 40% women don’t step out*. *They don’t go to the Anganwadi*. *But going to the home would be very inconvenient…*.*no*, *you cannot ask anyone [men] to come in [during a survey]*.”

*[Family member, FGD]*

## Discussion

Three overarching but interconnected findings in this study reflect deeply internalized social and gender norms that affect a mother’s ability to discuss gender-sensitive matters with a male interviewer in our study districts in Bihar: (1) mothers have limited agency to communicate with men about personal health issues, (2) gendered communication is influenced by restrictions of caste and religion, and (3) interviewer gender effects are likely associated with the survey topic.

Firstly, most mothers in our study exercised limited agency to communicate with any man (within the family or outside community members) about their personal health issues, even in the context of a household survey. They also lacked support within their families for crossing gender lines to discuss sensitive health issues. Regardless of age, caste and educational level, mothers were unable to talk to other men (aside from their husband) about female-specific topics. Other studies in India have shown that female modesty engrained in societal expectations linked to gender and social status make many women, especially younger women, reluctant to talk about any reproductive health matters with men, except professional health workers [16, 31, 32].

In traditional Indian settings, family and kinship structures usually determine what is considered to be acceptable and appropriate behaviour for most women. “Respectful avoidance” [16, 31, 32] is a prevailing social norm related to a woman’s social status. It is understood that a married woman should avoid speaking to any male relative who is the same age or older than her husband. In contrast, most women are able to have informal relationships with their husbands’ younger brothers. Thus, avoidance is considered appropriate behaviour for interactions with people of higher status which, in this case, means elder men in the family such as an elder brother-in-law and father-in-law. But women may engage freely with individuals of equal or lower status, such as a younger brother-in-law or other women in the family [16].

In our study area, communicating with a man may be akin to physical intimacy which is prohibited and unacceptable for a married woman; this reaction was also noted in an earlier study in southern India [32]. Given this context, if a mother is seen talking to a male interviewer, the scene can be easily misconstrued by onlookers leading to negative and even harmful consequences for the her. Most mothers in our study area preferred to avoid this situation. Mothers overwhelmingly preferred to discuss their personal health issues with a female interviewer as they perceived fewer risks if observed sharing information with an outside woman. These findings are consistent with other studies that discuss the restrictive gender roles in India regarding intra and extra-familial communication on health issues [31, 32].

Secondly, our findings draw attention to the ways in which gender, caste, religion and other social factors intersect to shape a mother’s everyday experiences with restrictions on communication and sharing information in our study districts. Upper and lower caste and Muslim mothers in our study face a different set of challenges regarding communication with a male interviewer. Of note is the difficulty in gaining entry into Hindu upper caste and Muslim households to interview mothers for survey research, and how this differs from access to mothers from the Hindu lower caste. We found that all mothers must be cautious in their interactions with men outside their family; however, this is especially salient for upper caste mothers. Social norms in India are more binding among upper caste women and failure to uphold them carries risk [33, 34]. Family honour is tightly tied to a woman’s behaviour which is especially true for the upper castes. Preserving the husband’s family honour as well as the outward appearance of respectability can significantly impact on a woman’s status and even her physical safety [31, 32]. It is not surprising that upper caste mothers in our study generally opposed any interaction with male enumerators whenever possible. Upper caste and religious constraints on a mother’s participation in a household survey, including strict observation of seclusion norms, could lead to selection bias in survey research through her self-exclusion. Fear of disapproval and opposition from peers and family may suppress the participation of upper caste and Muslim mothers, leading to a persistent lack of information about their health status and health care needs in survey data.

In contrast, lower caste mothers were more open to participating in a survey administered by a male interviewer. These women generally have more permissive families and greater mobility due to working as labourers [20]. While they may perceive fewer restrictions than upper caste mothers with regard to communicating with men outside of traditional family circles, they can face severe social hostility from upper caste communities for communicating across gender lines. Future studies may consider exploring the intersecting influences of caste, gender and other social factors (e.g., age and class) for a more nuanced understanding of interviewer effects.

Thirdly, we found certain interview topics were more likely to be susceptible to interviewer gender effects when included in a household survey, including questions about pregnancy or ANC, reproductive choices, breastfeeding, sexual behaviour and STIs. Mothers are willing to respond to these types of questions but only to a female enumerator. The only man with whom they can safely discuss these topics is their husband. In contrast, topics related to children’s health have no such restrictions. Mothers were willing and able to share health information about their children with interviewers of either gender. They generally experienced favourable social support within their family and community for crossing gender lines to discuss children’s health issues. Concern for their children’s wellbeing outweighs the potential downsides related to perceived disrespect and shame from being seen talking to an outside man. This may be less pronounced for upper caste mothers who prefer to avoid male interviewers altogether, regardless of the topic.

These findings from our qualitative research help us to understand the different results produced by the two probability household surveys. It is plausible that in these surveys, both under- and overestimated coverage reflect mothers’ attempts at survey avoidance due to the pressures of gender norms and social constraints. Low coverage for an indicator is usually interpreted to mean poor uptake of the particular service by the target population. However, lower coverage in this case may be due to the mother’s attempt to avoid a question or to her refusal to respond to the male interviewer in the NGO survey. This may be due to mothers’ unwillingness and inability (low agency) to respond accurately to questions posed by a male interviewer. In a survey, a refusal to answer a question typically results in a negative response, which reduces the coverage proportion for an indicator. Likewise, skipped questions often generate a cascading series of negative responses which again can artificially reduce the indicator value for that particular service. Alternatively, non-sampling error due to interviewer gender may reflect a situation where husbands (or other male family members) respond on behalf of their wives and offer inaccurate information in either direction (positive or negative responses to questions).

Nevertheless, interviewer gender effects do not always lead to underreporting due to negative responses. Avoidance can also occur when a mother responds YES in an attempt to end the survey quickly, which leads to higher counts resulting in erroneously overestimated coverage estimates. While we cannot know *a priori* which way the effects will go, the clear discordance of results in Table 2 suggest survey data in Aurangabad and Gopalganj districts may be unreliable when collected by a male enumerator for the reasons already outlined. Unreliable data due to effects of the interviewer-respondent relationship is a pattern seen in other methodological work investigating the role of the interviewer in household surveys [6].

Similar mixed findings have been reported in studies in other non-Western settings. For example, a case study of interviewers for a family planning intervention in Ghana showed differential responses given by women and men in surveys due an interviewer’s gender; responses concerned knowledge and use of contraceptives and the frequency of sexual activity [35]. A Nigerian study in Muslim neighbourhoods noted that a male member of the family was always present when the interviewer was a man which inhibited a woman’s answers to questions about family planning. Another Nigerian study also revealed discordant responses to questions on knowledge and use of contraception with male versus female interviewers; errors were bi-directional [36]. A study in Ghana cautioned against predicting the direction of response bias due to interviewer-gender effects [37]. That study compared survey responses to male and female interviewers and found differences in responses to knowledge and attitude questions regarding HIV/AIDS; however, there were few discrepancies in sexual behaviour reported to male enumerators. In contrast, a study in Zambia found, female respondents were more likely to report sexual behaviours to female interviewers than to males [11].

Our findings are consistent with these examples and underscore the likelihood that interviewer gender can introduce non-sampling error, and bi-directional error in household surveys with MNCH topics in traditional settings such as Aurangabad and Gopalganj. When respondents refuse to answer or they answer but respond incorrectly, the survey conclusions will be erroneous. Health policy decisions based on unreliable or inaccurate data may be misguided as a result.

### Strengths and limitations

This study triangulated perceptions of interviewer gender effects from several different angles including mothers, family members, FLW and survey interviewers themselves. We attempted to include both male and female interviewers; however, we were unable to interview any of the male enumerators from the NGO that conducted the survey in 2016. As a proxy, we interviewed male supervisors who were involved in the 2016 study and who had also previously worked as enumerators of various household surveys in Aurangabad and Gopalganj districts.

We found very few studies that considered the operational challenges of hiring female interviewers for household surveys. Two recent articles discussed the challenges of using female interviewers for surveys in Muslim countries [38, 39]. Both studies cited religious or cultural reasons that prevent women from travelling alone or entering the homes of strangers. Strategies for recruiting women as interviewers, such as hiring additional field staff to accompany the female interviewers, were found to increase the budget or complicate the study logistics. It is possible that similar challenges may be relevant to the locations in our study. However, in our own experience in the two study districts, we did not encounter any operational difficulties in hiring or training a team of qualified female interviewers. Some women may have declined to participate in interview teams if they were required to travel alone or ride a motorbike; however, these are logistical issues that were easy to address. Our female interviewers travelled in pairs along with a supervisor (usually male) for data collection. The female teams used mobile phones for data collection and to coordinate interview locations in each village which allowed the survey to move along quickly while ensuring high quality data.

This study makes three contributions. Firstly, this study contributes to the very limited qualitative research on the role of interviewer influence in household surveys. Secondly, it highlights perceptions from multiple perspectives (mothers, influential family members, frontline workers, and interviewers) about gender sensitivities to an interviewer’s gender. Thirdly, our findings extend the debate on interviewer gender effects to the Indian subcontinent and we empirically demonstrate discordance in survey results associated with interviewer gender across a wide range of indicators and social status.

## Conclusions

This study explores gender-specific constraints to participation in household surveys in Aurangabad and Gopalganj districts and how these constraints can affect the interpretation and accuracy of survey findings on MNCH indicators. Our findings highlight the influence of the interviewer-respondent relationship in the traditional and hierarchical setting of northern India with a culture of strict observance of social restrictions linked to gender and caste. Researchers need to be aware of how the interviewer-respondent dyad influences the capturing of survey data and biases that can result due to social distances.

In this case, social norms and gender biases in our study districts may have affected whether standard questions used in household surveys accurately measured coverage of topics ranging from childhood immunization to gender-sensitive services such as family planning, ANC and delivery care. Interviewer gender is a likely source of non-sampling error in household surveys when a male interviewer gathers data from a female respondent about gender sensitive issue. This factor may be particularly true in a traditional setting such as Aurangabad and Gopalganj.

Given the challenges of capturing accurate data through face-to-face surveys, research teams can take preventative measures to mitigate the potential impact of interviewer characteristics, in particular when designing protocols and questionnaires for maternal and reproductive health studies and when selecting and training the data collection team. Our findings highlight the importance of training enumerators to establish trust and confidence and to enquire when obtaining “informed consent” if the female or male respondent views specific categories of survey questions as culturally sensitive. We also suggest screening interviewer candidates for gender bias prior to hiring them for a household survey. For example, this can be done during the recruitment process by asking male and female interviewer candidates about their own preferences for an interviewer’s gender, if they are uncomfortable interviewing respondents of either gender and if they have any particular beliefs or preconceptions about gender roles. While this type of interviewer screening may be an afterthought in some analyses, we suggest it as a standard operating procedure. Similarly, using qualitative methods as part of the survey design stage will help reveal sociocultural norms of the study setting that may help avoid biased results in the larger survey.

Social distance between interviewer and respondent can be minimized in household surveys on MNCH topics carried out in a traditional Indian context like rural Bihar. Practical actions include matching interviewer and respondent characteristics, especially gender and caste/religion, and when not possible, randomizing interviewer characteristics across respondents. This advisory may vary outside of South Asia. Female interviewers should be considered an optimal choice for areas such as Aurangabad and Gopalganj districts to facilitate access to mothers, reduce discomfort during the interview process and obtain better quality data with more accurate and consistent responses to sensitive topics. MNCH surveys in settings such as our study districts that consistently use all male interviewers, when gender effects are possible, may result in bias; when repeated across studies this bias can alter our understanding of public health issues and affect policy decisions. Misguided policy and programme responses can occur as a consequence of biased survey data. Future research may consider applying an intersectionality framework to analyse the influence of gender, caste, religion, age and other social factors on survey research in rural India. Intersectionality can help us to understand how multiple factors can overlap and combine to result in different types of restrictions or discrimination experienced by female respondents.

This research provides context for the unexplained differences in the quantitative results from the twin surveys carried out in Aurangabad and Gopalganj in 2016. While we add to the methodological research on the interviewer-respondent dyad in India, our findings have implications for elsewhere. These results raise serious issues for protocol design for household survey research in any traditional setting where women’s autonomy is limited and suggest the need for additional research systematising the effect of gender when measuring population health. Systematic examination of the potential for interviewer-gender effects and a better understanding of the sociocultural norms are needed in different contexts where surveys take place.

## Supporting information

S1 File Data collection tools. Focus group discussion guides. (a) Mothers of Children < 5 Years. (b) Household members (husbands, grandparents, others living in households with mothers of children < 5 years). Individual interview guides. (a) ASHA/AWW. (b) Interviewers from prior surveys in Bihar.
(DOCX)

S1 Table Summary of final themes in the Bihar dataset. (DOCX)
