# Supplementary material for: Does interviewer gender influence a mother’s response to household surveys about maternal and child health in traditional settings? A qualitative study in Bihar, India
Source: PLoS One. 2021 Jun 16;16(6):e0252120. doi: 10.1371/journal.pone.0252120 (PMC8208568; doi:10.1371/journal.pone.0252120)
Supplement: S1 File — Focus group discussion guides. (a) Mothers of Children < 5 Years. (b) Household members (husbands, grandparents, others living in households with mothers of children < 5 years). Individual interview guides. (a) ASHA/AWW. (b) Interviewers from prior surveys in Bihar. (DOCX) [file pone.0252120.s001.docx]

**S1 File:**

**Data Collection Tools**

[Focus Group Discussion Guide 2](#_Toc69213462)

[Mothers of Children < 5 Years 2](#_Toc69213463)

[Household members (husbands, grandparents, others living in households with mothers of children < 5 years) 4](#_Toc69213464)

[Individual Interview Guide 6](#_Toc69213465)

[ASHA/AWW 6](#_Toc69213466)

[Interviewers from prior surveys in Bihar 8](#_Toc69213467)

# Focus Group Discussion Guide

## Mothers of Children < 5 Years

**Basic Profile:**

| Respondent code |  |
| --- | --- |
| Location |  |
| Age |  |
| Education |  |
| Religion |  |
| Caste |  |

1. In your household, is it acceptable for you to discuss your personal health issues with other family members or people living in your household?

- With whom? Why is it acceptable to discuss with these family or household members?
- Why do you feel it is not acceptable?

1. Is acceptable for you to discuss your personal health issues with people who do not live in your household?

- With whom? Why do you feel it is acceptable to discuss with these people?
- Why do you feel it is not acceptable?

1. Now, switching to your children’s health -- do you feel it is acceptable for you to discuss your children’s health issues with other family members or people living in your household?

- With whom? Why is it acceptable to discuss with these family or household members?
- Why do you feel it is not acceptable?

1. Do you feel it is acceptable for you to discuss your children’s health issues with people who do not live in your household?

- With whom? Why do you feel it is acceptable to discuss with these people?
- Why is this not acceptable?

1. If a female interviewer came to your house during a regular household survey, would you feel comfortable answering questions about your personal health?

- What makes you feel comfortable? Uncomfortable?

1. Would you feel comfortable answering questions about your health to a male interviewer at your house?

- What makes you feel comfortable? Uncomfortable?

1. What would matter the most to you in terms of how you answer questions regarding your health? (This question will use a listing/ranking exercise)

- Would the location of the interview matter to you?
- Would the interviewer’s age matter to you?
- Their gender?
- Their social position/work function?
- Presence of other people in the house at the time of an interview?
- Anything else you can think of?

1. Are there any specific health topics you would not like to discuss with a female interviewer? With a male interviewer? (This question will use a matrix ranking exercise)

- What about seeking antenatal care?
- Birth preparedness?
- Family planning methods?
- Immunization status of your children?
- Nutrition of your children?
- FLW support?
- Other topics you can think of?

1. Would you feel expected to answer a certain way to certain questions? Which types of questions? Can you explain?
2. When household surveys are carried out in your community, are the interviewers usually male or female? Can either type of interviewer do the job equally well? If there is a difference can you explain?

**Focus Group Discussion Guide**

## Household members (husbands, grandparents, others living in households with mothers of children < 5 years)

**Basic Profile:**

| Respondent code |  |
| --- | --- |
| Location |  |
| Gender |  |
| Age |  |
| Education |  |
| Religion |  |
| Caste |  |

1. In your household, is it acceptable for a mother to discuss her personal health issues with other family members or people living in the household?

- With whom? Why is it acceptable to discuss with these family or household members?
- Why do you feel it is not acceptable?

1. Is acceptable for a mother to discuss her personal health issues with people who do not live in the household?

- With whom? Why do you feel it is acceptable to discuss with these people?
- Why do you feel it is not acceptable?

1. Now, switching to children’s health -- do you feel it is acceptable for a mother to discuss her children’s health issues with other family members or people living in the household?

- With whom? Why is it acceptable to discuss with these family or household members?
- Why do you feel it is not acceptable?

1. Do you feel it is acceptable for a mother to discuss her children’s health issues with people who do not live in the household?

- With whom? Why
- Why is this not acceptable?

1. If a female interviewer came to your house during a regular household survey, would a mother feel comfortable answering questions about her personal health? Uncomfortable? Why?

- Would the interview be acceptable to you? Unacceptable? Why?

1. Would a mother feel comfortable answering questions about her health to a male interviewer at your house? Uncomfortable? Why?

- Would the interview be acceptable to you? Unacceptable? Why?

1. What do you think influences how a mother answers survey questions about her health?

(This question will use a listing/ranking exercise)

- Would the location of the interview matter to her?
- Would the interviewer’s age matter to her?
- Their gender?
- Their social position/work function?
- Presence of other people in the house at the time of an interview?
- Anything else you can think of?

1. Are there any specific health topics that are not acceptable for a mother to discuss with a female interviewer in this community? With a male interviewer? (This question will use a matrix ranking exercise)

- What about seeking antenatal care?
- Birth preparedness?
- Family planning methods?
- Immunization status of children?
- Nutrition of children?
- FLW support?
- Other topics you can think of?

1. Are mothers expected to answer a certain way to certain questions? Which types of questions? Can you explain?
2. When household surveys are carried out in your community, are the interviewers usually male or female? Can either type of interviewer do the job equally well? If there is a difference can you explain?

# Individual Interview Guide

## ASHA/AWW

**Basic Profile:**

| Respondent code |  |
| --- | --- |
| Location |  |
| Gender |  |
| Age |  |
| Education |  |
| Religion |  |
| Caste |  |

1. In this community, do you feel it is acceptable for a mother to discuss her personal health issues with other family members or people living in her household?

- With whom? Why is it acceptable to discuss with these family or household members?
- Why do you feel it is not acceptable?

1. Do you feel it is acceptable for a mother to discuss her personal health issues with people who do not live in her household?

- With whom? Why do you feel it is acceptable to discuss with these people?
- Why do you feel it is not acceptable?

1. Now, switching to the topic of children’s’ health -- do you feel it is acceptable for a mother to discuss her children’s health issues with other family members or people living in the same household?

- With whom? Why is it acceptable to discuss with these family or household members?
- Why do you feel it is not acceptable?

1. Do you feel it is acceptable for a mother to discuss her children’s health issues with people who do not live in the same household?

- With whom? Why do you feel it is acceptable to discuss with these people?
- Why is this not acceptable?

1. Do you think a mother would feel comfortable answering questions about her personal health to a female interviewer during a regular household survey? Why or why not?

- In your opinion, is the interview is acceptable? Unacceptable? Why?

1. Do you think a mother would feel comfortable answering questions about her personal health to a male interviewer during a regular household survey? Why or why not?

In your opinion, is the interview is acceptable? Unacceptable? Why?

1. What do you think influences how a mother answers survey questions about her health?

- Would the location of the interview matter to her?
- Would the interviewer’s age matter to her?
- Their gender?
- Their social position/work function?
- Presence of other people in the house at the time of an interview?
- Anything else you can think of?

1. In your opinion, are there any specific health topics that are not acceptable for a mother to discuss with a female interviewer in this community? With a male interviewer?

- What about seeking antenatal care?
- Birth preparedness?
- Family planning methods?
- Immunization status of children?
- Nutrition of children?
- FLW support?
- Other topics you can think of?

1. In your opinion, are mothers expected to answer a certain way to certain questions? Which types of questions? Can you explain?
2. When household surveys are carried out in your community, are the interviewers usually male or female? Can either type of interviewer do the job equally well? If there is a difference can you explain?

**Individual Interview Guide**

## Interviewers from prior surveys in Bihar

**Basic Profile:**

| Respondent code |  |
| --- | --- |
| Location |  |
| Gender |  |
| Age |  |
| Education |  |
| Religion |  |
| Caste |  |

1. Can you recall the LQAS survey that you participated in September 2016 in XXXXX districts?
2. Did you carry out the interviews by yourself or in a team? If team, please elaborate whether you asked all of the questions yourself or did someone else assist you? Were you trained on how to administer the questionnaires? Did you feel comfortable with all of the questions on the questionnaire? Please explain.
3. In general, were mothers from all religions equally comfortable in participating in the survey. Please explain your response. Similarly, did mothers from all caste communities seem comfortable in participating in the survey, please explain your response?
4. Where did you usually carry out the survey (inside the house, outside, nearby)? Please explain.
5. Before the interviews, did mothers or the family members ask questions to you? What kind of questions, please elaborate? Did the family’s religion or caste make any difference and how?
6. During and after the interview, did mothers or the family members ask any questions to you? What kind of questions, please elaborate? Did the family’s religion or caste make any difference and how?
7. Where you able to interact with all mothers that you approached? Were there any houses where you were not allowed to meet the mothers? If yes, then why? Did the family’s religion or caste make any difference and how?
8. Do you think or have you come across any household where women were not allowed to go out of the household? Please elaborate? Did the family’s religion or caste make any difference and how?
9. Were there any questions mothers seemed comfortable answering?

- PROBE on which topics and why.
- Did the mother’s religion, caste, or education make any difference?

1. Were there any questions mothers seemed uncomfortable answering?

- PROBE on which topics and why mothers felt discomfort.
- Did the mother’s religion, caste, or education make any difference?

1. Were there certain topics mothers tended not to answer at all (or skip)?

- PROBE on which topics and why it seemed mothers would avoid these.
- Did the mother’s religion, caste, or education make any difference?

1. In your past experience as an enumerator, did you ever face any hesitation or resistance on the part of the family to approach a mother from any religious community for the survey? Please elaborate either way.
2. In your past experience as an enumerator, did you ever face any hesitation or resistance on the part of the family to approach a mother from any caste community for the survey? Please elaborate either way.
3. In your experience, were you able to approach a mother from all religious and caste community directly for the interview? Did you need permission from a family member, please explain? Which family member? Can a female enumerator approach a mother from all religious and caste freely or does she need permission?
4. In your experience, who signed the consent form for the survey? In your opinion, was taking consent from mother for the survey, was different or similar in all religious and caste communities, please explain your response?
5. In your opinion, are there any specific health topics that are not acceptable for a mother to discuss with a female interviewer in this community? With a male interviewer? PROBE specific topics (seeking antenatal care, birth preparedness, family planning methods, breastfeeding, menstruation, immunization status of children, nutrition of children, sexually transmitted diseases, FLW support to mother, other topics)
6. In your opinion, are mothers from any religious community expected to answer a certain way to certain questions? Which types of questions? Can you explain?
7. In your opinion, are mothers from any caste community expected to answer a certain way to certain questions? Which types of questions? Can you explain?
8. When household surveys are carried out in this community, are the interviewers usually male or female? In your opinion, can either type of interviewer do the job equally well? If there is a difference can you explain?
