# Supplementary material for: Does interviewer gender influence a mother’s response to household surveys about maternal and child health in traditional settings? A qualitative study in Bihar, India
Source: PLoS One. 2021 Jun 16;16(6):e0252120. doi: 10.1371/journal.pone.0252120 (PMC8208568; doi:10.1371/journal.pone.0252120)
Supplement: S1 Table — (DOCX) [file pone.0252120.s002.docx]

S1 Table. Summary of final themes in the Bihar dataset

| Themes | Categories | Illustrative quotes |
| --- | --- | --- |
| Social expectations around communication and gender roles | Communication and deference in married household | *“The custom in Bihar is that women don’t speak… not with her male relatives. With us, there are some hierarchies, okay, which we have to adhere to.”* |
|  | Seclusion norms | *‘‘It is our traditional value that women show respect by distancing themselves from other ‘outside’ men and this must be adhered to.”* |
|  | Sense of shame | *“There is no sense of shame if a woman is talking to another woman. But women in the village will definitely feel ashamed while talking to men.”* |
|  | Fear of consequences | *“Women do not talk to men because they fear that they will later be questioned and harassed by family or community members if found speaking to men.”* |
|  | Role of female interviewer | *“For conducting health surveys, women will talk to other women openly. If a man comes then I can't talk to him... When it comes to talking to a woman there will be a free conversation and I can share everything they are like our sisters.”* |
| Acceptability of survey topics | Verbal and non-verbal resistance of the family | *“Family planning proved to be a little difficult with Muslim and lower caste families…With hindu upper caste families there were permission or consent related issues.”* |
|  | Gender preferences linked to survey topics | *“While talking to men [interviewer] we have to think a lot before we speak. If they are asking about the child's health, then she [the mother] will talk with them but when it comes to talk about her own health problems, she won't talk due to hesitation and shame.”* |
|  | Unrestricted topics | *“When it comes to vaccinations for children, women can talk to both men and women about that, but they can’t talk about their own health related problems with men.”* |
|  | Restricted topics | *“On ANC, birth preparedness, family planning, breastfeeding, menstruation, STIs [sexually transmitted diseases]: If a man is asking the questions, they would have problems, it would be easier for women…irrespective of the caste or religion.”* |
| Intersection of gender, caste and religion | Mobility constraints | *“In Hindus, upper caste households they don’t get permission to go outside unless for an emergency. In Muslim houses it’s the same …it happens here, but in lower castes there’s no restriction, she can go anywhere.”* |
|  | Communication constraints | *“Mothers don’t have the freedom to talk to new people…In villages, even when they are educated, they behave like this, this is the culture. It’s in every community, but in upper castes this is more so the case.”* |
|  | Age-related constraints | *“There were instances in which newly married and new mothers were not allowed to do the interviews, mostly in upper caste families….yes, in all [upper] castes, Rajputs, Brahmins, Srivastavas, even getting a child vaccinated, the mothers-in-law get it done, the new mothers aren’t allowed to step out…. mostly with women aged 22-28 years.”* |
|  | Permissions from family | *“With the general and upper castes, Muslims, and actually with people who were more educated, there were more permissions needed. With lower caste families there was no issue, they [mothers] spoke easily.”* |
| Other factors that influence survey participation | Mothers’ perspective | *“If the male enumerator is an educated like you, my husband will allow me to talk but if there is a less educated male enumerator, my husband will start asking me questions. But, if there is a female enumerator, he will have no problem in sending me.”* |
|  | Family perspective | *“First of all, women should be available for women to be comfortable. Everyone agrees. Education comes second… Then place and the presence or absence of other people. The place where there are no men is the best place [for an interview] ...Other people should not be there. The Anganwadi centre is the best, it’s the most convenient.”* |
